# Antagonistic Interactions and Clutch-Dependent Sensitivity Induce Variable Responses to Ocean Acidification and Warming in Squid (*Doryteuthis pealeii*) Embryos and Paralarvae

**DOI:** 10.3389/fphys.2020.00501

**Published:** 2020-05-20

**Authors:** Casey J. Zakroff, T. Aran Mooney

**Affiliations:** ^1^Biology Department, Woods Hole Oceanographic Institution, Woods Hole, MA, United States; ^2^Massachusetts Institute of Technology–Woods Hole Oceanographic Institution Joint Program in Oceanography/Applied Ocean Science and Engineering, Cambridge, MA, United States

**Keywords:** cephalopod, hypercapnia, Myopsida, temperature, stress, multifactor, malformation

## Abstract

Ocean acidification (OA) and warming seas are significant concerns for coastal systems and species. The Atlantic longfin squid, *Doryteuthis pealeii*, a core component of the Northwest Atlantic trophic web, has demonstrated impacts, such as reduced growth and delayed development, under high chronic exposure to acidification (2200 ppm), but the combined effects of OA and warming have not been explored in this species. In this study, *D. pealeii* egg capsules were reared under a combination of several acidification levels (400, 2200, and 3500 ppm) and temperatures (20 and 27°C). Hatchlings were measured for a range of metrics [dorsal mantle length (DML), yolk sac volume (YV), malformation, and hatching success] in three trials over the 2016 breeding season (May – October). Although notable resistance to stressors was seen, highlighting variability within and between clutches, reduced DML and malformation of the embryos occurred at the highest OA exposure. Surprisingly, increased temperatures did not appear to exacerbate OA impacts, although responses were variable. Time to hatching, which increased with acidification, decreased much more drastically under warming and, further, decreased or removed delays caused by acidification. Hatching success, while variable by clutch, showed consistent patterns of greater late stage loss of embryos under acidification and greater early stage loss under warming, highlighting the potential difference in timing between these stressors for this system, i.e., that acidification stress builds up and causes impacts over time within the egg capsule as the embryos grow and respire. High OA-exposed hatchlings from the warmer conditions often showed reduced impacts compared to those reared in ambient temperatures. This may be due to the increased developmental rate and subsequently reduced OA exposure time of embryos in the higher temperature treatment. These results indicate a substantive potential plasticity to multiple stressors during the embryonic development of this species of squid, but do not predict how this species would fare under these future ocean scenarios.

## Introduction

Coastal ecosystems are seeing levels of acidification and warming today that are not predicted for the open ocean for hundreds of years (Pachauri and Meyer, 2014). OA can be enhanced in coastal regions due to increased pH variability from freshwater influx, urbanization, and pollution (Gledhill et al., 2015). Rapid coastal warming, particularly in ocean warming hotspots like the northwest Atlantic and the Gulf of Maine region, is already causing substantive impacts to vital marine services and valuable fisheries (Hobday and Pecl, 2014; Pershing et al., 2015; Saba et al., 2016). The complexity of this scenario is further compounded by the potential for interactive effects, whether additive, synergistic, or antagonistic, between these stressors for a range of potentially sensitive organisms and processes (Crain et al., 2008; Kroeker et al., 2013; Breitburg et al., 2015). Further, it is becoming clear that while data in global change is limited and generalizations are to some degree necessary, many organismal responses to multistressor scenarios are context specific to population and/or region (Kroeker et al., 2017). Life stage specific responses, for example, particularly for early development and dispersal where sensitivities are often thought to be highest, are foundational to understanding how populations may be impacted under multistressor scenarios (Byrne, 2011; Haigh et al., 2015).

Coastal squids, the Myopsid squids, are a fundamental component of shelf and nearshore food webs, and the longfin inshore squid, *Doryteuthis pealeii*, serves this role along the northwest Atlantic shelf system (Jacobson, 2005). These squid also support a valuable fishery in New England, with landings of 11,000 mt in 2018 (NOAA, 2019). These benthopelagic squid overwinter in the deeper, warmer waters of the shelf, but from May to October they aggregate in the shallow nearshore along the northwest Atlantic coastline to breed (Jacobson, 2005). They lay their eggs in mucous-bound capsules (tens to hundreds of eggs per capsule), which are tied to benthic structures or the substrate, often in large masses consisting of many capsules (Shashar and Hanlon, 2013). These eggs are stationary and must develop under whatever environmental conditions and variability they are exposed. Observers have recorded eggs laid at depths of up to 50 m, in temperatures of 10–23°C, and salinities of 30–32, which contextualizes a presumed preferred laying habitat for this species (McMahon and Summers, 1971; Jacobson, 2005). Less is known about the preferences for pH of egg laying habitat for this species, but pH_t_ calculated from shelf carbonate system profiles throughout their habitat range indicate a typical exposure range of 7.88–8.2 (600–250 ppm pCO_2_) during the breeding season (Wang et al., 2013; Zakroff et al., 2019). Navarro et al. (2018) reported preferred egg laying habitat for the California market squid, *Doryteuthis opalescens*, as requiring pH_t_ greater than 7.8 and O_2_ concentrations greater than 160 μmol with no apparent temperature limitation within the region. These static ranges do not account for the possible variability of temperature and carbonate system measures across temporal scales, as have been observed in this nearshore system, but at least provide a framework for ideal tolerance windows (Hong et al., 2009; Connolly and Lentz, 2014).

Developing embryos are often thought to be particularly sensitive to added stressors because they have limited available energy stores and are actively building the machinery needed to maintain homeostasis (Steer et al., 2004; Hu et al., 2010). This paradigm is challenged, however, by the inherent need for coastal embryos to cope with a highly variable environment and the potential plasticity of an embryo given active and adaptive developmental pathways (Hamdoun and Epel, 2007). The question, then, becomes one of the limits of resilience and plasticity during embryonic development, and when human-driven global ocean change will push systems past those limits.

A growing body of research has examined the impacts of various stressors on the early life stages of Myopsid squid. Egg capsules of *D. opalescens* reared under acidification (pH 7.57, pCO_2_ ∼ 1440 ppm) and hypoxia (80 μM O_2_) showed delays to development, potential reductions in YV, and decreases in statolith size relative to the embryo (Navarro et al., 2016). Pimentel et al. (2012) exposed egg capsules of the European market squid, *Loligo vulgaris*, to warming (+2°C above regional seasonal averages) and described a 28-fold increase in oxygen consumption during embryogenesis, resulting in rapid depletion of available oxygen and causing metabolic suppression in late stage embryos. Further work with individualized *L. vulgaris* embryos (embryos removed from the protections of the egg capsule) under +2°C warming resulted in metabolic suppression that encouraged premature hatching, with an increase in malformations observed among the hatched paralarvae (Rosa et al., 2012). This study also noted that warming-exposed paralarvae, as opposed to encapsulated embryos, activated an integrated stress response of heat shock proteins and antioxidant enzymes, suggesting the transition to planktonic life could come with the addition of a more robust stress response toolbox (Rosa et al., 2012; Robin et al., 2014). Few studies of warming and acidification have been performed with Myopsid squid embryos. Rosa et al. (2014) exposed seasonal clutches of individualized *L. vulgaris* embryos to the combined effects of warming and acidification (+2°C and pCO_2_ ∼ 1650 ppm) and observed decreased hatching success (47%) and increased premature hatching and abnormalities, most strongly within the summer clutch. They also observed delays in development time and decreases in oxygen consumption under acidification that were antagonistic to the increases in these rates caused by warming. The effects of individualization, removing squid eggs from the environmental protections imbued by the egg capsule during embryonic development, have not been determined.

Despite their ecological and economic importance, we know little regarding the interactive effects of OA and temperature on *D. pealeii.* Studies with this species have only focused on acidification. Kaplan et al. (2013); experiments ran in 2011 reared *D. pealeii* egg capsules under a low, facility ambient (550 ppm), and high acidification (2200 ppm), observing development delays of about 1 day, a decrease in DML of the hatchling paralarvae, and decreased size and quality of the statoliths. Subsequently, Zakroff et al. (2019; experiments ran in 2013) expanded upon this preliminary work, exposing *D. pealeii* egg capsules to a range of pCO_2_ levels (from 400 to 2200 ppm), noting delays in development time, decreases in DML, and smaller, rougher statoliths that indicated a potential dose response threshold of 1300 ppm. In addition, this study highlighted notable variability in response intensity across the breeding season, demonstrating potentially different physiological strategies or responses to acidification stress within the egg capsules that expressed as different levels of sensitivity or resistance across the season (Zakroff et al., 2019). Behavioral experiments run in parallel to and following Zakroff et al. (2019) showed full years where paralarvae showed little to no response to acidification even up to the original 2200 ppm level, suggesting greater levels of resilience than had been expected (Zakroff et al., 2018; experiments ran from 2013 to 2015).

In this study, we examined the extent of stress resistance and response variability in developing *D. pealeii* embryos by both increasing the acidification exposure (2200 ppm was considered a variable response, while 3500 ppm was used as a positive control) and by adding warming (+2°C above peak breeding season temperature for Vineyard Sound, MA, United States) as a potentially compounding stressor. Metrics analyzed are the same as those in Zakroff et al. (2019) except that proportions of malformation in the paralarvae were added based on their prevalence in work by Rosa et al. (2012, 2014) and statolith data are not included to maintain concision in this manuscript. Further, unlike most of the previously cited work, which used wild collected or lab produced egg capsules most likely sourced from multiple parents, egg capsule maternity was monitored, allowing for an examination of both between and within clutch variability of stress responses.

## Materials and Methods

Experiments were performed between May and October of 2016 at the Environmental Systems Laboratory (ESL) of the Woods Hole Oceanographic Institution, Woods Hole, MA, United States during peak breeding season of *Doryteuthis pealeii* for this region (Arnold et al., 1974; Jacobson, 2005). Methods presented throughout are similar to those reported in detail in Zakroff et al. (2019) so will be reiterated in brief, but with differences noted.

### Squid Capture and Care

Squid were acquired from the Marine Biological Laboratory (MBL) Marine Resources Center from trawls performed at 10–30 m depth in Vineyard Sound at the Menemsha Bight of Martha’s Vineyard. Squid were either selected on ship or following offloading from the ship, but prior to deposition into holding aquaria at the MBL. Females of 15–25 cm DML that exhibited the least signs of stress (calmly resting at bottom or gently hovering with no damage or lesions to fins or skin) and had bright orange accessory nidamental glands were selected. Per trip, three adult females were hand-selected from the catch and each carefully placed into their own seawater filled cooler. The squid were then driven quickly and gently to the ESL. Each female squid was gently transferred from the coolers into one of three flow-through round tanks (120 cm in diameter, 70 cm depth). Overall, time from capture to introduction to the tanks at the ESL was less than 6 h from capture to tank.

Each female had her own tank with no males or other squid present. These tanks were fed by Vineyard Sound seawater that had been sand-filtered and cooled to 15°C (Salinity = 32, pH_nbs_ = 7.96). As noted in Zakroff et al. (2019) this temperature occurs during the breeding season (9.60–25.40°C from May to October 2016, Station BZBM3, US DOC/NOAA/NWS/NDBC > National Data Buoy Center), but is typically lower than the ambient mean (19.57°C from May to October 2016, Station BZBM3, US DOC/NOAA/NWS/NDBC > National Data Buoy Center), and was used to avoid increased damage and stress due to increased metabolism and activity under higher temperatures. Each aquarium had a ca. 2 cm thick layer of sand at the bottom, was continuously bubbled with air, and was covered throughout the day to avoid startling. Each tank included false egg capsules, comprised of the inflated fingers of seawater soaked and cleaned nitrile gloves zip-tied to a weight, to encourage egg-laying on a clean surface away from the substrate or air hose. Squid were fed once per day with local killifish, *Fundulus heteroclitus*. All female squid were fed and maintained in the ESL until they died following egg laying.

New female squid were brought in for each trial. Female squid typically laid egg capsules after 2 days of capture, producing small mucous-bound masses of around 2–30 egg capsules, with each capsule containing 80–300 eggs. These female squid fertilized eggs with stored sperm from breeding that occurred prior to capture, so paternity was unknown (and potentially complex; Buresch et al., 2001), but maternity of all eggs was known. Tanks were checked for eggs each morning. If eggs were present in any single tank, they were immediately hand-transferred into a clean 5-gallon bucket of 15°C, filtered seawater and taken to the room containing the egg culturing system. Only egg capsules of high quality (orange-tinted, thin, and oblong fingers with no notable air pockets or other damages) were selected and randomly hand sorted into the cups of the experimental system to initiate a trial (described below). Only one trial was run at a time, so the eggs used were always from a single female/tank, and had been laid the night prior to discovery and introduction to the system. No additional eggs, laid by the same squid or other females of the same catch, were collected for any trial of the experiments described here.

### Squid Egg Culture System: Acidification and Warming

Details of the culture and acidification system are the same as those reported in Zakroff et al. (2019). In brief, 15°C, 10 μm filtered, and UV-treated Vineyard Sound water was fed into an air-bubbled header tank, which gravity-fed three H-shaped PVC equilibration chambers that each contained four airstones (two per leg) bubbling with gas mixtures for each acidification treatment line (400, 2200, and 3500 ppm CO_2_). Treatment gases were not tested with a CO_2_ analyzer as they were in Zakroff et al. (2019) because treatments exceeded the range of the meter. Water in the ESL increases in pCO_2_ compared to environmental ambient, from 400 ppm to about 550 ppm, so the water in the 400 ppm control was first degassed with N_2_ in one H-chamber and then re-equilibrated with ambient air in two additional, subsequent chambers. Given the several stages of storage and filtration input water goes through as it enters both the ESL and the acidification system, it is presumed that it is not subject to small-scale environmental variability due to mixing and integration of water over time. No such variability was noted in temperature, salinity, or pH, but moderate variability in alkalinity was observed within and between trials (Table 1).

**TABLE 1:** Seawater chemistry, maternal wet weight, number of egg capsules, and number of paralarvae subsampled for each treatment of each trial.

**Laying date**	**Mother wet weight (g)**	**Temp (°C)**	**Treatment pCO_2_ (ppm)**	**# Egg capsules**	**pH_total_**	**Salinity**	**A_T_ (mmol kgSW^–1^)**	**Ω_Arag_**	**pC0_2_ (ppm)**	**n**	
										DML	YV
June 19	44.5	20.86	400	3	7.98 (0.02)	32.82 (0.16)	2222.6 (45.6)	2.44 (0.12)	474.96 (17.7)	94	96
			2200	3	7.41 (0.02)	32.84 (0.19)	2236.6 (37.1)	0.76 (0.04)	2005.07 (53.2)	59	80
			3500	2	7.25 (0.02)	32.87 (0.17)	2201.2 (60.1)	0.53 (0.04)	2922.38 (133.7)	89	30
		27.04	400	3	7.93 (0.04)	32.94 (0.14)	2172.1 (16.3)	2.72 (0.19)	523.64 (55.4)	105	107
			2200	3	7.41 (0.03)	33.01 (0.12)	2241.6 (36.5)	0.96 (0.07)	2092.08 (137.0)	93	58
			3500	2	7.26 (0.03)	32.99 (0.17)	2185.0 (15.0)	0.68 (0.04)	2917.18 (207.0)	59	38
July 28	56.5	19.86	400	4	8.00 (0.04)	33.19 (0.17)	2143.5 (7.77)	2.40 (0.21)	426.05 (46.3)	115	111
			2200	4	7.43 (0.03)	33.23 (0.32)	2154.4 (22.2)	0.73 (0.07)	1856.25 (138.9)	93	104
			3500	3	7.26 (0.04)	33.11 (0.18)	2146.0 (13.9)	0.52 (0.06)	2729.6 (290.0)	91	48
		27.26	400	3	7.93 (0.06)	33.53 (0.16)	2147.8 (25.2)	2.76 (0.33)	512.67 (85.9)	45	28
			2200	3	7.41 (0.04)	33.45 (0.20)	2146.6 (14.5)	0.95 (0.09)	1985.6 (187.7)	101	54
			3500	3	7.25 (0.04)	33.42 (0.14)	2140.0 (13.5)	0.67 (0.06)	2889.3 (289.2)	58	28
September 14	62.5	19.46	400	3	8.03 (0.03)	33.33 (0.18)	2099.7 (19.4)	2.44 (0.11)	387.25 (28.3)	94	90
			2200	3	7.41 (0.02)	33.60 (0.16)	2126.0 (16.2)	0.69 (0.02)	1908.70 (88.8)	59	57
			3500	3	7.26 (0.01)	33.48 (0.11)	2116.1 (11.9)	0.50 (0.02)	2687.75 (89.1)	89	67
		27.47	400	3	7.97 (0.03)	33.83 (0.15)	2093.65 (15.0)	2.87 (0.19)	452.46 (41.6)	105	100
			2200	3	7.42 (0.02)	33.78 (0.14)	2119.13 (9.8)	0.96 (0.05)	1907.1 (108.2)	93	85
			3500	3	7.28 (0.02)	33.97 (0.43)	2154.9 (76.9)	0.73 (0.06)	2691.1 (86.9)	59	51

Acidified water left the equilibration chambers and entered PVC manifolds and was carried by drip lines to the individual experimental culture cups (pre-soaked, 1-L PET containers, Solo Foodservice, Lake Forest, IL, United States; 5 cups per treatment ^∗^ 3 treatments per water bath = 15 cups per water bath). Each cup was sealed with a lid through which a drip line was fed to the bottom of the cup. A bubbling line of the appropriate gas concentration was also fed through the lid to about half way up the cup and aligned underneath the outflow window, so as to not disturb the egg capsule during development, but push hatched paralarvae away from the outflow screen. Water outflowed through a 5 μm mesh window in the treatment cups into the surrounding water bath, which then outflowed to the drain.

Each water bath was maintained at 15°C by an aquarium chiller (Oceanic Aquarium Chiller 1/10hp, Oceanic Systems, Walnut Creek, CA, United States) and heaters (JÄGER 3603, EHEIM GmbH and Co., Deizisau, DE) to match the maternal holding tanks until introduction of eggs for a trial. Although in Zakroff et al. (2019) temperature acclimation between holding tanks and experimental tanks was not performed, as transfer from 15 to 20°C showed no impact to the eggs, the shock from 15 to 27°C was highly impactful to egg capsule survival in preliminary experiments and so methods were changed to acclimate eggs slowly to temperature. Upon introduction of eggs, water baths were increased in temperature 1 degree every 2 h until desired treatments temperatures were reached: 20°C, the average seasonal temperature, and 27°C, two degrees above peak temperature (25°C) in Vineyard Sound from May to October (2011–2016, Station BZBM3, US DOC/NOAA/NWS/NDBC > National Data Buoy Center). Water bath temperatures were swapped, the 20°C bath changed to 27°C and vice versa, between trials in order to reduce any potential impact of water bath or position in the room on the temperature treatments. Each water bath was monitored with a HOBO data logger (HOBO pendant model UA-004-64, Onset Data Loggers, Bourne, MA, United States), which recorded temperature and ambient light every 15 min. The culture room used ceiling mounted fluorescent lighting, which was set to a 14:10 light:dark photoperiod (broadly that of the natural system during this time).

### An Experimental Trial: Egg Rearing and Monitoring

Trials were initiated by the presence of eggs in one of the maternal holding tanks and were demarcated by lay date (June 19, July 28, and September 14). Egg capsules were randomly sorted by hand: one capsule each into four out of the five cups in each treatment (with the last cup acting as an abiotic control for monitoring of seawater chemistry). A “full” trial would therefore be comprised of 12 egg capsules per water bath (four capsules, one per cup, in each of the three acidification treatments), requiring 24 capsules for the two temperature treatments/water baths. This “full” number of egg capsules was not always reached in each trial, so egg capsules were sorted to prioritize each treatment having as many treatment replicates as possible (see number of egg capsules in Table 1).

Following the introduction of eggs and temperature acclimation, water samples were taken from every cup for carbonate system measurements. These methods mirror exactly those described in Zakroff et al. (2019) except that salinity was no longer taken with bottle samples and was instead measured using a salinity probe (Orion Star^TM^ A329, Thermo Fisher Scientific Inc., Waltham, MA, United States). Data from spectrophotometric pH_t_, alkalinity, salinity, and temperature were input into CO2SYS (Pierrot et al., 2006), calculated with dissociation constants from Mehrbach et al. (1973) and sulfate constants from Dickson (1990) to produce pCO_2_ values for the seawater treatments (Table 1). After a trial’s initiation, these measurements were performed weekly on the abiotic control cup (twice more, usually). The pH_nbs_ of all cups was measured every 3 days using a three-point standard calibrated pH probe (Orion Star^TM^ A329, Thermo Fisher Scientific Inc., Waltham, MA, United States). These pH measurements were used primarily to monitor the stability of the pH in the system and ensure pH of the biotic cups did not vary notably from the abiotic controls.

Egg capsules were left to develop undisturbed, with particular care taken during chemical monitoring, within the treatment system. Cups were checked daily to observe development and check for hatchlings. Under ambient pCO_2_, hatching typically initiated after 13–15 days in the 20°C temperature control, and 8–10 days in the 27°C warming treatment. Each hatching day, all paralarvae were removed, counted, and subsampled for the various measurements described below. All the paralarvae that were not subsampled for analysis were anesthetized with 7.5% w/v MgCl_2_ mixed with equal part seawater and preserved in 70% ethanol in microcentrifuge tubes (0.65 mL and 1.7 mL Costar microcentrifuge tubes, Corning, Inc., Corning, NY, United States). Handling and preservation of subsampled paralarvae is described below. No hatched squid remained in the cups across days, so all paralarvae included in the data are from their day of hatching (less than 1 day old).

### Water Quality

Water chemistry, particularly temperature, salinity, and pH_t_, were quite stable within and between experiments (Table 1). Seawater alkalinity varied the most in this system, which may have contributed to the variability of the pCO_2_ equilibrations. Within a treatment, pH_t_ and calculated pCO_2_ were consistent across cups (KW, *p* > 0.05 for all treatments in all trials). Input gas mixtures were the same across trials, but resultant pCO_2_ equilibrations were variable between temperature treatments and across trials (Table 1). This variability was most likely due to the flow-through nature of the system and fluctuations in seawater input flow rates, although it may also represent some uncontrolled for seawater variability due to the natural sourcing of water from Vineyard Sound. Equilibrations at higher CO_2_ concentrations were much more challenging to maintain, resulting in seawater pCO_2_ values somewhat lower than the input gas concentrations (e.g., 2729.6 ppm for the 20°C × 3500 ppm treatment in the July 28 trial; Table 1). Despite these variations, data are reported across trials by the input gas concentrations for concision and clarity. However, it should be understood that these three concentrations, 400, 2200, and 3500 ppm, are acting more as a negative control, variable response level, and positive control, respectively, across these experiments rather than a precise representation of response at that equilibrated seawater CO_2_ concentration.

### Metrics

**FIGURE 1:**
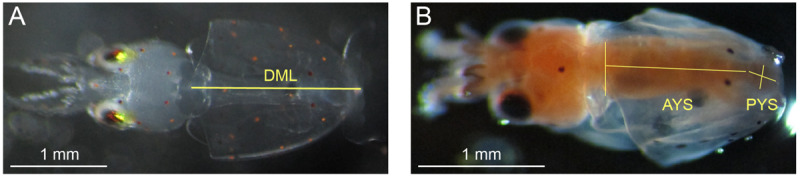
Morphometrics measured on *Doryteuthis pealeii* paralarvae through microscope imagery. **(A)** Dorsal mantle length (DML; the superimposed yellow line) of an anesthetized paralarvae. **(B)** Squid paralarvae were fixed and stained with oil red O for measurement of YV. The length and width of both the anterior yolk sac (AYS), which was modeled as either a cone or cylinder (cone, in this case), and the posterior yolk sac (PYS), which was modeled as an ellipsoid were measured (superimposed yellow lines). Each image has a unique 1 mm white scale bar at bottom left.

Methods for measurements of DML and YV were the same as those reported in Zakroff et al. (2019). In brief, paralarvae anesthetized in 7.5% w/v MgCl_2_ in equal parts seawater (around 10 per treatment for the first 4 days of hatching) were photographed under dissecting scope (SteREO Discovery.V8, Carl Zeiss AG, Oberkochen, DE) and measured for DML using ImageJ (National Institutes of Health, Rockville, MD, United States; Figure 1). Paralarvae measured for DML were preserved in ethanol with the rest of the day’s hatch. YV was measured by anesthetizing, fixing, and staining paralarvae with oil red O following Gallager et al. (1986) and processing in ImageJ (Figure 1) following the methods of Vidal et al. (2002a). No premature paralarvae, those with external yolk remaining, were included in either the DML or YV datasets.

Hatching time and success likewise were as described in Zakroff et al. (2019). The process of counting and preserving hatchling paralarvae continued each day until 2 days with no paralarvae found in the cup was reached. The egg capsule would then be removed, photographed, and dissected under dissecting scope. The remaining unhatched embryos were counted and categorized by simple visual discrimination of their stage of development (early: stages 1–16, middle: stages 17–26, and late: stages 27–30) adapted from Arnold et al. (1974).

### Malformation

On the day with the greatest proportion of hatching for each cup, a random subsample of around 50 paralarvae were taken and categorized for malformations (sample sizes varied depending on hatching dynamics, but only samples of 20 paralarvae or more were used in the analysis). The subsampled paralarvae were categorized as either *Normal*: showing no external yolk or malformations, *Premature*: showing external yolk remaining post-hatch, but no other notable malformations, *Eye Bulge*: showing an inflation of the membrane around the eyes, or *Malformed Head*, showing a misshapen, often pointed or oblong head, occasionally also with odd growths or a malformed mantle.

### Statistics

Statistical analyses were run in a Jupyter Notebook (Project Jupyter) using Python (version 3.5.5, Python Software Foundation). Data were first tested for normality with Shapiro–Wilks tests (*p* > 0.05) and through assessment of the linearity of quantile plots and the shapes of histograms. DML data are reported as means ± one standard deviation, primarily for easier relation to their visualizations. Log-transformed YV data are reported as the back transformed mean and values ± one standard deviation. Parametric data are often presented with a LR trend line, but these are not presented for statistical power; they serve as visual aids of trends.

Normally distributed data (DML and log-transformed YV) were then processed for group differences of means with multi-factor Type II ANOVAs. For the ANOVA model, pCO_2_, temperature, and trial were all treated as independent factors. Trials were intended to serve as experimental replicates, however, variability in response, likely caused by parentage and seasonality, interferes with this assumption. Replicate trials independent of external influences were not achievable for us for this organism and experimental system. Experimental cups serve as treatment replicates within a trial. Since a single egg capsule was used per cup, the effects of cup versus capsule (represented by number of eggs) variability cannot be disentangled statistically and are not included in the ANOVA’s discussed here (data was therefore compiled across cups; an egg number ANOVA is discussed below). ANOVA data are presented with calculated effect sizes (ω^2^). A Tukey’s HSD *post hoc* test was used to determine which groups showed statistically significant (*p* < 0.05) differences.

Non-parametric water quality data were analyzed with Kruskal–Wallis (KW) tests for difference between treatments. Non-parametric distributional data (hatching time curves, hatching success, and malformation) were analyzed using *G*-tests and are described for trends with LR, though the statistical power of these regressions is low due to low sample size (data for these metrics is per cup/egg capsule; maximum *n* = 4; Table 1).

### Assumptions

Although each trial in this experiment contains eggs from a single, separate mother, since replicates of different mothers were not run at or very near the same time point, the effects of maternity and seasonality could not be disentangled in our data. Maternal wet weight is noted in Table 1, but is not included in statistical models, as both the low sample size of mothers and the experimental design did not allow for it to be statistically distinguishable from trial effects.

Number of embryos within an egg capsule was noted in Zakroff et al. (2019) as a potentially impactful continuous variable on the state of hatched paralarvae, particularly for DML. As each cup contained only a single egg capsule in these experiments, any effects particular to the culture cup could not be disentangled from effects of egg capsule (or number of eggs per capsule). Type II ANOVA’s were run with number of eggs per capsule as a continuous covariate to examine the potential impact of cup/number of eggs per capsule on response to acidification and warming.

On top of trial and cup effects, Zakroff et al. (2019) highlighted different responses to stressors across the days of the squid eggs hatching. While responses likely change across hatching days here as well, digging into them is beyond the scope of this manuscript. Further, samples were taken over fewer days of hatching in this dataset (4 days compared to six) making the dataset less robust for that type of analysis. Statistical models are presented with the assumption that effects of hatching date are occurring, but can be ignored in order to investigate the overall impacts of the stressors.

## Results

### Dorsal Mantle Length

**FIGURE 2:**
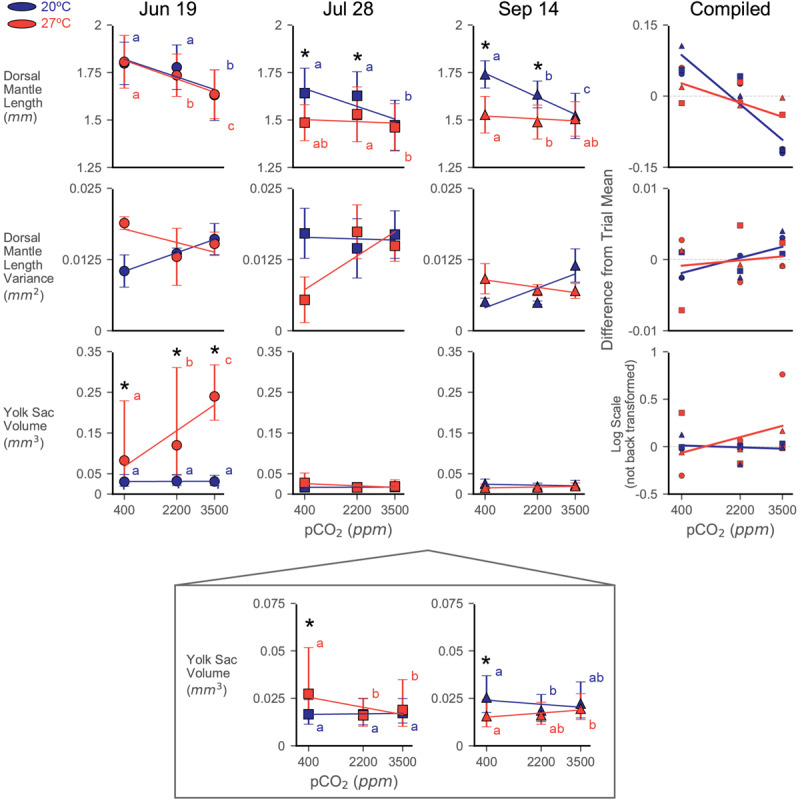
Dorsal mantle length, its variance, and YV data from three experiments rearing squid eggs under acidification (in parts per million CO_2_; *x*-axis) and warming (color; blue/dark = 20°C, red/light = 27°C). Yolk data were transformed to logarithmic scale for statistical analyses and has been back transformed for the depiction of trial data. A zoom box is provided beneath the July 28 and September 14 YV plot in order to see statistical difference and trends. Symbols represent trial, demarcated by dates eggs were laid (titles). Symbols depict means with error bars of one standard deviation. Letters indicate statistical groups across acidification levels within a temperature treatment from a Tukey’s HSD. Asterisks indicate statistically significant differences between temperature treatments at the same acidification level. Regression lines are presented primarily as an aid to visualizing trends in the data and are not intended to indicate statistical power. The compiled plots show data from all trials normalized by subtracting the average value for a trial from its data; relative differences in yolk data have not been back transformed from the logarithmic scale. Error bars have been removed and symbols shrunk in order to emphasize trendlines.

Dorsal mantle length of the paralarvae was impacted by both acidification and warming, but responses varied substantially between trials (Figure 2). Control treatment (20°C × 400 ppm) DML shifted across trials similar to the pattern reported in Zakroff et al. (2019). Notably, this pattern of seasonal DML shift appears unrelated to maternal weight: DML started around the typical paralarval size of 1.8 mm (June 19, 44.5 g mother: 1.80 ± 0.11 mm), reached its minimum at the peak of summer (July 28, 56.5 g mother: 1.64 ± 0.13 mm), and then increased again (September 14, 62.5 g mother: 1.74 ± 0.07 mm). Compiled across trials, the data indicate that while interactions between all factors are significant, the individual factors of trial (ω^2^ = 0.257), pCO_2_ (ω^2^ = 0.119), and temperature (ω^2^ = 0.054) had the greatest effects on DML (Supplementary Table S1). Significant, relatively strong effects of acidification in ANOVA’s and LR’s across all trials are driven primarily by the consistently strong response at the 3500 ppm positive control (Figure 2). Assessments of responses to acidification were therefore focused on results from the 2200 ppm treatment.

Paralarvae from the June 19 trial were resistant to both stressors in terms of DML, only showing a notable decrease in size at the 3500 ppm (20°C: 1.63 ± 0.13 mm; 27°C: 1.64 ± 0.13 mm) positive control acidification level (Figure 2 and Supplementary Table S1). The 2200 ppm exposed paralarvae from the 27°C treatment (1.74 ± 0.11 mm) showed a slight decrease relative to their acidification control (1.81 ± 0.14 mm), but were not different from the 2200 ppm from the 20°C water bath (1.78 ± 0.12 mm; Figure 2). Interactions between pCO_2_ and temperature were not significant in this trial (Table 1).

The July 28 trial showed a substantial response to temperature in the DML data (decreasing to 1.49 ± 0.10 mm at the 27°C × 400 ppm treatment), but no effect of acidification at the 2200 ppm level (Supplementary Table S1 and Figure 2). The 3500 ppm positive control resulted in pCO_2_ having the greatest effect size in this trial (ω^2^ = 0.132), but temperature was nearly as impactful (ω^2^ = 0.077), and these stressors appeared to interact slightly (ω^2^ = 0.029, Supplementary Table S1).

Decreases in DML were seen in the September 14 trial with both acidification at the 2200 ppm treatment (20°C: 1.63 ± 0.07) and warming (27°C × 400 ppm: 1.53 ± 0.10; 27°C × 2200 ppm: 1.49 ± 0.09; Figure 2). Both acidification (ω^2^ = 0.165) and warming (ω^2^ = 0.252) had significant impacts on DML, as did their interaction (ω^2^ = 0.090), which was the largest of all the trials (Supplementary Table S1).

Notably, warming did not simply transpose the acidification impact downward or exacerbate the slope/severity of acidification effects (Figure 2). Rather, in trials where warming had a significant effect (July 28 and September 14), acidification impacts in the warming treatment were decreased (e.g., order of magnitude decrease in slope in September 14; 20°C LR: −6.97^∗^10^–5^, 27°C LR: −8.10^∗^10^–6^). In the compiled data, this results in a shift from a significant decrease with increasing acidification (20°C, LR, slope = −5.75^∗^10^–5^, *R*^2^ = 0.824, *p* < 0.001) to a slight decrease with increase acidification under warming (27°C, LR, slope = −2.27^∗^10^–5^, *R*^2^ = 0.406, *p* = 0.065; Figure 2).

### Variance of DML Data

Variance in DML showed broadly similar patterns between the June 19 and September 14 trials, with variance increasing with acidification at 20°C and decreasing with acidification at 27°C, while the July 28 trial showed the opposite trends (Figure 2). As a result, the compiled data show a weak increasing trend with acidification at 20°C (LR, slope = 1.19^∗^10^–6^, *R*^2^ = 0.445, *p* = 0.057) that diminishes to roughly flat line at 27°C (LR, slope = 4.19^∗^10^–7^, *R*^2^ = 0.025, *p* = 0.682; Figure 2). Individual paired *t*-tests of variance between treatments broadly showed no significant changes in DML variance [two-sample *t*(2), *p* > 0.05 for most treatment pairings within in each trial], except in the September 14, 20°C × 400 ppm vs. 20°C × 3500 ppm test [two-sample *t*(2) = −2.96, *p* = 0.042], although these results are likely impacted by low sample sizes (Table 1: number of egg capsules per treatment).

**FIGURE 3:**
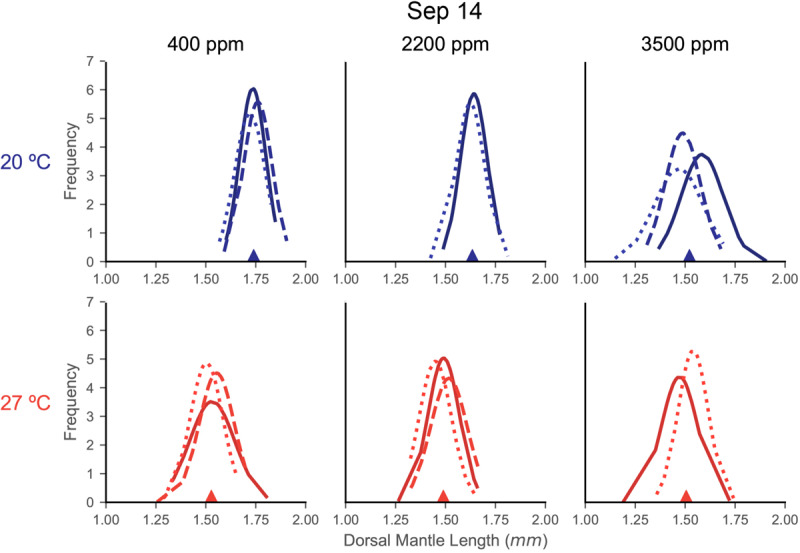
Fitted normal distributions of DML histogram density from individual egg capsules in each treatment of the September 14 trial. Histograms are not shown for clarity of curves, but DML data were segmented in 0.05 mm bins. Each plot is a treatment combination, with column determining acidification (titles) and row determining temperature treatment (also differentiated by color: top/blue = 20°C, bottom/red = 27°C). Each line represents the curve from the sampling of an individual egg capsule for DML (*x*-axis). Lines are shaded and patterned to help differentiate individual egg capsules within a plot, but this carries no relationship or meaning across the plots. The filled triangle on the *x*-axis marks the mean value for the compiled sample of that treatment.

Distributions of DML for each capsule within a treatment were relatively similar in shape, indicating consistency in responses among the egg capsules of a mother’s clutch (September 14: Figure 3; June 19: Supplementary Figure S1; July 28: Supplementary Figure S2). In the September 14 trial, where DML was sensitive to both stressors, egg capsule distribution demonstrated wider spread with decreased peaks under warming (Figure 3). At 20°C, acidification caused September 14 egg capsules to translated to decreased sizes at 2200 ppm, but distributions retained the same shape, before flattening, spreading, and become more varied at 3500 ppm (Figure 3).

### Yolk Sac Volume

Yolk sac volume responses appear to have been consistently affected (compiled data Type II ANOVA, *p* < 0.001 for all factors; Supplementary Table S1) by both temperature (ω^2^ = 0.045) and pCO_2_ (ω^2^ = 0.008), but the direction and intensity of those responses shift strongly between trials (ω^2^ = 0.383, Figure 2), particularly due to the interaction between trial and warming response (ω^2^ = 0.153). Control treatment hatchling YV followed a similar pattern as DML across trials, decreasing to its minimum in the July 28 trial (20°C × 400 ppm: June 19, 0.030 mm^3^ [0.019–0.048 mm^3^]; July 28, 0.017 mm^3^ [0.011–0.024 mm^3^]; September 14, 0.025 mm^3^ [0.018–0.037 mm^3^]).

In the June 19 trial, warming appeared to have the most substantial effect (ω^2^ = 0.410; Supplementary Table S1) on remaining paralarval yolk reserves, which increased under warming and increased further under combined warming and acidification (Figure 2). Paralarvae reared at 3500 ppm in the 20°C water bath hatched with internal YV of 0.031 mm^3^ (0.021–0.046 mm^3^), similar to the control, while YV of those in the 27°C water bath were 0.240 mm^3^ (0.181–0.317 mm^3^).

For the July 28 trial, while all factors were significant (Supplementary Table S1), the interaction between warming and acidification had the greatest impact (ω^2^ = 0.045) on paralarval YV. Similar to the June 19 trial, acidification had no notable effect on YV in the 20°C water bath and warming increased (though by much less than in June 19) remaining YV at the 400 ppm treatment (0.027 mm^3^ [0.014–0.052 mm^3^]). In contrast to June 19, however, YV in the 27°C water bath decreased with increasing acidification in this trial (3500 ppm, 0.019 mm^3^ [0.010–0.035 mm^3^]; Figure 2).

Paralarvae in the September 14 clutch showed weak, but significant, overall responses in YV, with temperature having the greatest effect (ω^2^ = 0.109; Supplementary Table S1). Unlike the other trials, the September 14 paralarvae showed a slight decrease in hatching YV with increasing acidification in the 20°C water bath (Figure 2). Also unique to the September 14 trial, warming to 27°C resulted in paralarvae hatched with less YV at the 400 ppm treatment (0.016 mm^3^ [0.010 mm^3^–0.024 mm^3^]). Increasing acidification resulted in slightly increased remnant YV in the 27°C water bath, similar to, but much weaker than, the June 19 data (Figure 2).

Patterns of YV variance were inconsistent across trials and broadly showed no notable trends across acidification [two-sample *t*(2), *p* > 0.05 for most treatment pairings within in each trial]. A significant decrease in YV variance was seen in the 27°C water bath of the June 19 paralarvae between both the 400 ppm [1.044 ± 0.079 mm^6^; two-sample *t*(2) = 13.30, *p* < 0.001] and 2200 ppm [0.935 ± 0.061 mm^6^; two-sample *t*(2) = 13.92, *p* = 0.005] treatments and the 3500 ppm sample (0.083 ± 0.007 mm^6^).

### Comparing DML and YV

**FIGURE 4:**
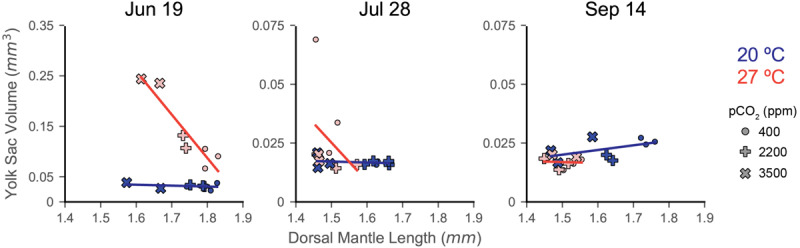
Response of individual egg capsules within a clutch to acidification and warming as indicated by comparing YV (*y*-axis; note shift in scale from June 19 to July 28) with DML (*x*-axis). Trials are indicated by title. Symbol shape indicates acidification treatment (circle = 400 ppm, plus sign = 2200 ppm, *X* = 3500 ppm), while color denotes temperature (blue/dark = 20°C; red/light = 27°C). Symbols represent the means for a single egg capsule (one cup within the experimental system). No error bars are depicted for visual clarity. Trend lines are regressions of each temperature treatment, but are only presented as visual aides.

In order to investigate clutch-specific patterns of physiological response to both acidification and warming stress, YV was plotted against DML for each egg capsule of each treatment (Figure 4). The 27°C treatment of the June 19 showed the strongest trend (LR, slope = −8.60, *R*^2^ = 0.865, *p* = 0.002; Figure 4), with warming and acidification having resulted in smaller paralarvae with less consumed yolk before hatching. The July 28 eggs, conversely, showed a weak trend of smaller paralarvae hatching with more yolk consumed under the same conditions. The September 14 clutch demonstrates a much weaker trend under both acidification and warming, but of a similar response type to the June 19 clutch. This trial also differs by having the only positive slope of the 20°C exposures (LR, slope = 0.020), with paralarvae having hatched smaller and with less yolk under increased acidification (Figure 4).

### Egg Number

Type II ANOVA’s were run with number of eggs per capsule as an independent continuous covariate, but did not have a significant effect on DML or YV in these experiments (multi-factor Type II ANOVA, *p* > 0.05 for all trials and in combined data). For this reason, this factor was not included in the model and statistics and these data not presented.

### Hatching Time

**FIGURE 5:**
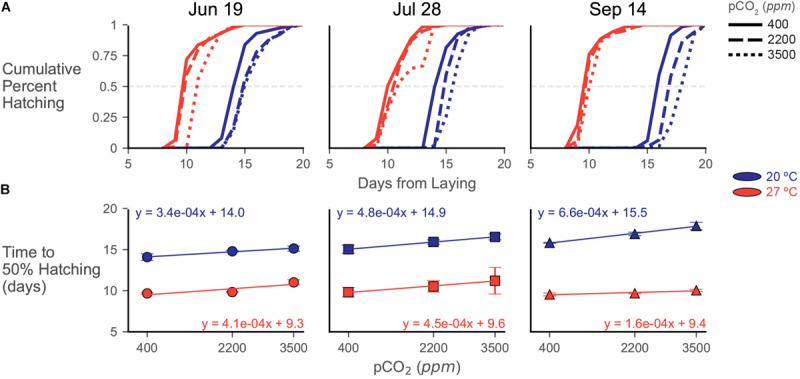
Hatching data from experiments depicted as **(A)** cumulative hatching curves and **(B)** plots of time to 50% hatch. **(A)** Cumulative hatching curves are shown for each trial (lay date in title). Patterning of lines represents acidification treatments (smaller hash = more acidic), while color represents temperature treatment (blue/dark = 20°C, red/light = 27°C). Error bars/shading omitted for visual clarity of the curves. The gray dashed line represents the 50% hatching mark. **(B)** Time to 50% hatching data across acidification exposures, calculated from the curves in **(A)**, plotted for each trial. Symbols depict means (and represent trial) with errorbars of one standard deviation. Color represents temperature treatment as in **(A)**. Regression lines with related equations are presented to visualize trends and assess changes in slope and y-intercept across treatments and trials.

Increased temperature increased the rate of embryonic development, resulting in 27°C egg capsules consistently hatching sooner (around 9 days) than their 20°C counterparts (around 14–15 days) in all trials (Figure 5A). While time to hatching increased for the 20°C treatments across the breeding season, as was seen in Zakroff et al. (2019); this seasonal increase to hatching time disappears in the 27°C treatments (see y-intercepts in Figure 5B). Increasing acidification broadly delayed hatching by around 1.5 days, but these impacts were somewhat dampened by warming, although responses to combined stressors varied across trials (see slopes in Figure 5B).

In the June 19 eggs, time to 50% hatching was delayed in the 20°C treatment from 14.09 ± 0.40 days at 400 ppm to 14.79 ± 0.10 days at 2200 ppm and 15.13 ± 0.32 days at 3500 ppm (Figure 5B). Hatching distributions were significantly different between the 400 ppm and both increased acidification treatments [*G*(7), *p* << 0.001 for both pairs] at this temperature, but the 2200 and 3500 ppm curves were not statistically distinct [*G*(6) = 4.699, *p* = 0.583]. In the 27°C treatment, time to 50% hatching was delayed from 9.67 ± 0.08 days at 400 ppm and 9.83 ± 0.05 days at 2200 ppm to 11.01 ± 0.32 days at 3500 ppm CO_2_. At this temperature, all hatching curves were different from each other [*G*(7), *p* << 0.001 for all pCO_2_ treatment pairs], but the differences between 400 and 2200 ppm were two orders of magnitude lower (G statistic of around 50 compared to around 1000) than pairings with the 3500 ppm treatment.

Delays in hatching occurred more consistently and progressively with increasing acidification in the July 28 trial (Figure 5). Within each temperature treatment, each hatching distribution at each pCO_2_ treatment was significantly different from each other [20°C, *G*(6), *p* << 0.001 and 27°C, *G*(11), *p* << 0.001 for all pCO_2_ treatment pairs]. Time to 50% hatching increased at 20°C from 15.04 ± 0.49 days at 400 ppm to 15.92 ± 0.27 days at 2200 ppm and 16.54 ± 0.36 days at 3500 ppm. At 27°C, 50% hatching was delayed from 9.81 ± 0.59 days, to 10.52 ± 0.70 days, then to 11.22 ± 1.62 days with increasing acidification (Figure 5B).

While hatching was clearly delayed in the 20°C water bath of the September 14 trial, acidification responses were strongly dampened at 27°C (Figure 5). Distributions of cumulative percent hatching were statistically distinct in both the 20°C [*G*(6), *p* << 0.001 for all pCO_2_ treatment pairs] and 27°C [*G*(11), *p* << 0.001 for all pCO_2_ treatment pairs] water baths, but the differences (as assessed by G statistics and p values) are an order of magnitude higher in the 20°C samples. Time to 50% hatching had the greatest delay in the 20°C samples of the September 14 trial, increasing from 15.83 ± 0.06 days at 400 ppm to 17.87 ± 0.452 at 3500 ppm (Figure 5B). Contrastingly, this trial also had the smallest delay in its 27°C samples, increasing from 9.54 ± 0.18 days to 10.04 ± 0.14 days.

### Hatching Success

**FIGURE 6:**
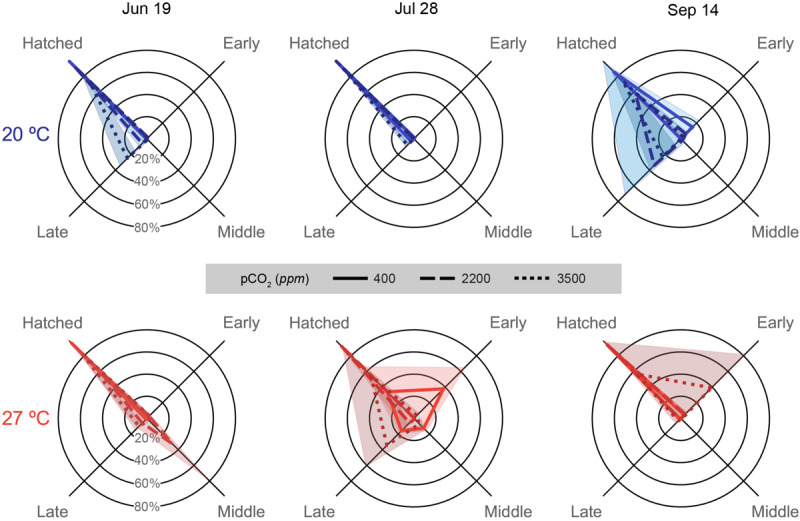
Embryonic survival data depicted for each trial (column title) on spider plots: axes represent categorical variables, while rings represent proportions of embryos in each category (labeled in June 19 plots). Categories include, clockwise from top left, Hatched: embryos that successfully hatched from the egg capsules (includes premature and malformed hatchlings), and embryos that ceased development either Early: Arnold stages 1–16, Middle: Arnold stages 17–26, or Late: Arnold stages 27–30 (Arnold et al., 1974). Lines represent means, while shading represents one standard deviation. Line patterns and shade of color represent acidification treatments (smaller hash/darker color = more acidic). Plot position and data coloration represent temperature treatment (top/blue = 20°C, bottom/red = 27°C).

Hatching success decreased both with acidification and warming, with increased acidification typically resulting more late stage losses, while warming resulted in more early to middle stage losses (Figure 6). Response patterns in hatching success were unique in each trial, as with previous metrics.

In the July 19 trial, hatching was quite high in the 400 (98.9 ± 1.0%) and 2200 ppm (92.7 ± 3.9%) treatments of the 20°C and the 400 ppm at 27°C (96.1 ± 2.9%). Distributions of staged failed embryos and hatched paralarvae were significantly different for all pCO_2_ treatment combinations within each temperature [*G*(3), *p* < 0.001 for all pCO_2_ treatment pairs] and for all pCO_2_ comparisons across temperatures [*G*(3), *p* < 0.001 for comparison of 2200 and 3500 ppm across temperatures] except at 400 ppm [*G*(3) = 5.313, *p* = 0.150; Supplementary Table S2]. The 2200 ppm at 27°C sampled had a single egg capsule completely fail at the middle stages, which drove down over hatching success for the treatment (59.2 ± 42.2%). The 3500 ppm treatments at both temperatures (20°C: 73.2 ± 10.3%; 27°C: 84.1 ± 6.6%) had decreased hatching due to losses at late stages.

Egg capsules of the July 28 trial showed high hatching success across acidification treatments at 20°C (all above 90%; Supplementary Table S2), but had substantive decreases in the 400 (34.0 ± 31.9%) and 3500 ppm (54.7 ± 36.3%) treatments at 27°C. Embryos halted development in multiple egg capsules of the 400 ppm treatment at all stages, but mostly early, while late stage losses drove the decrease in hatching success at 3500 ppm (Supplementary Table S2). Hatching success distributions were all distinct for all treatments within this trial [*G*(3), *p* < 0.001 for almost all pCO_2_ and temperature treatment pairs; Supplementary Table S2], though the 400 and 3500 ppm treatments at 20°C were nearly the same due to the presence of slight late stage losses [*G*(3) = 10.77, *p* = 0.013].

Hatching success was highest in the 400 (94.0 ± 5.0%) and 2200 ppm (93.8 ± 2.8%) treatments at 27°C in the September 14 data (Supplementary Table S2), which showed very similar distributions with only slight early stage losses [*G*(3) = 3.524, *p* = 0.318]. All other treatment combinations showed significant differences in hatching success distribution within this trial [*G*(3), *p* < 0.001; Supplementary Table S2]. The 3500 ppm treatment at 27°C had low hatching success (56.8 ± 40.7%) driven by near complete early stage loss in a single capsule, as well as slight early stage losses in the other capsules. The 400 ppm treatment at 20°C had relatively high hatching success (85.1 ± 8.3%), although lower compared to the 27°C due to high early stage losses. Increased acidification at 20°C had much higher decreases in hatching success, however, due to large late stage losses in both the 2200 (57.8 ± 41.0%) and 3500 ppm (74.2 ± 15.0%) treatments.

### Malformation

**FIGURE 7:**
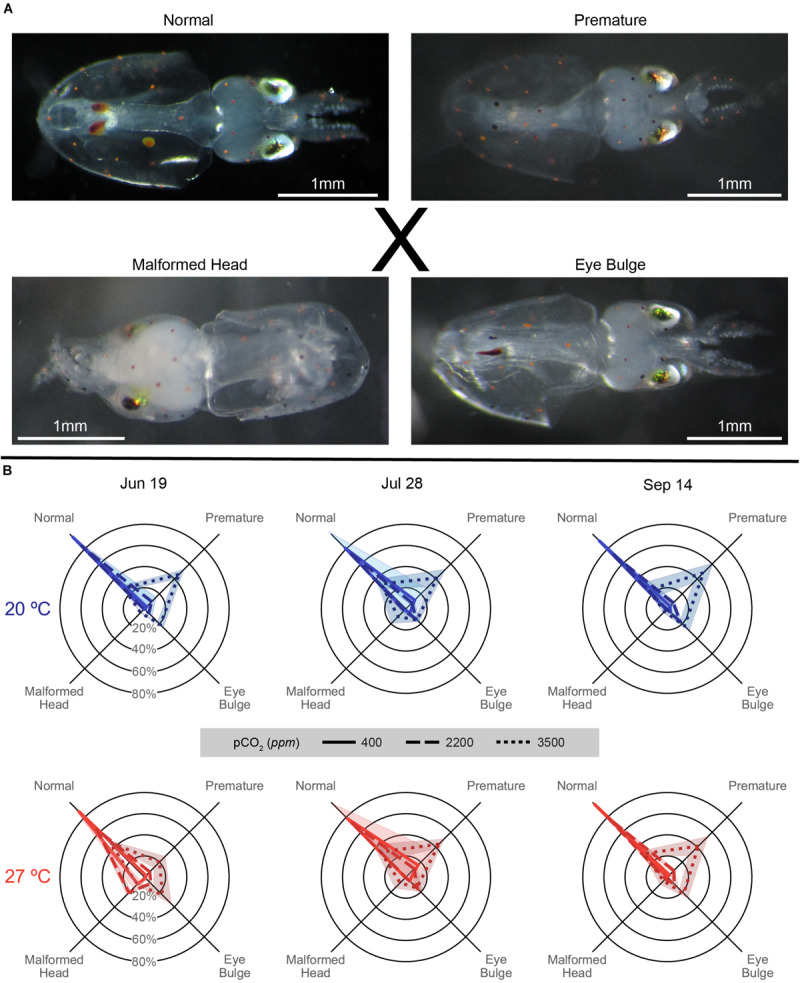
**(A)** Images of types of hatched *Doryteuthis pealeii* paralarvae corresponding to the axes categories in the data figure **(B)**, as referenced by the ‘X.’ From top left: Normal: a typical hatchling paralarvae, Premature: a paralarvae showing remaining external yolk, Eye Bulge: a paralarvae with inflation of the membrane around the eye, and Malformed Head: a paralarvae with misshapen head, can also present with strange growths or misshapen mantle. All images have a unique 1 mm white scale line in the bottom left. **(B)** Malformation data for each trial (column title) on spider plots: axes represent categorical variables, while rings represent proportions of embryos in each category (labeled in June 19 plots). Categories include, clockwise from top left, Normal, Premature, Eye Bulge, and Malformed Head as depicted in **(A)**. Lines represent means, while shading represents one standard deviation. Line patterns and shade of color represent acidification treatments (smaller hash/darker color = more acidic). Plot position and data coloration represent temperature treatment (top/blue = 20°C, bottom/red = 27°C).

The patterns of malformation were relatively consistent across trials, with increasing acidification producing greater proportions of premature and eye bulge paralarvae, while warming dampened the acidification impacts slightly and increased the proportion of malformed head paralarvae (Figure 7B). In all trials, proportionally less premature paralarvae were seen in the 3500 ppm treatment at 27°C than at 20°C (Supplementary Table S3). Malformation distributions compared across trials, showed significant shifts as a result of trial for the 20°C × 2200 ppm treatment and all 27°C treatments [*G*(6), *p* < 0.001 for listed treatments].

Paralarvae from the June 19 trial generally demonstrated the pattern described above, but with particularly notable increases in malformed head paralarvae in all acidification levels at 27°C (Figure 7 and Supplementary Table S3). All distributions in all within temperature and within pCO_2_ pairings in this trial significantly differed from each other [*G*(3), *p* < 0.01; Supplementary Table S3].

In the July 28 trial, paralarvae showed broadly similar patterns of malformation across temperatures, with acidification impacts being slightly more prominent in the 20°C and temperature impacts being minimal (in part possibly driven by sample size shifts between temperatures; Supplementary Table S3). Although all within-temperature comparisons of malformation distributions between pCO_2_ treatments were significant, those at 27°C were less different than those at 20°C [*G*(3), *p* ≤ 0.001; see exponents in Supplementary Table S3]. Notably, distributions of malformations between pCO_2_ treatments across temperatures were either comparatively weakly, in the case of 400 ppm [*G*(3) = 11.57, *p* = 0.009], or not significantly different, for 2200 [*G*(3) = 4.485, *p* = 0.214] and 3500 ppm [*G*(3) = 0.338, *p* = 0.953], in this trial.

The September 14 paralarvae also demonstrated more prominent acidification impacts at 20°C. These impacts were slightly dampened with warming, under which there only showed a slight, but weak, increase in malformed head proportions (Figure 7). Distributions of malformations were significantly different between all pCO_2_ treatment pairs within temperatures [*G*(3), *p* < 0.001; Supplementary Table S3] owing to increasing premature and eye bulge proportions with increasing acidification. Differences across temperatures, driven by the dampening and shifts described with warming above, were significant in the 400 ppm [*G*(3) = 16.32, *p* < 0.001] treatment and neared significance in the 2200 [*G*(3) = 6.622, *p* = 0.085] and 3500 ppm [*G*(3) = 6.829, *p* = 0.078] treatments.

## Discussion

These experiments demonstrated clutch-dependent sensitivity to the combination of high levels of acidification and warming stress in the egg capsules of *D. pealeii* across the 2016 breeding season. Embryos appear to be capable of developing normally, at least in terms of size, yolk consumption, and survival up to at least 2200 ppm CO_2_, a value which is not predicted in ‘no reduction of emissions’ scenarios in the open ocean until at least the year 2300 (Caldeira and Wickett, 2003). The consistent hatching delay, as well as the increased proportion of late stage loss and premature paralarvae observed, suggest that acidification may cause metabolic suppression, particularly late in development, as was described for *L. vulgaris* embryos under warming (Pimentel et al., 2012; Rosa et al., 2012). Metabolic suppression was long suggested as an expected impact of OA on squid because squid hemocyanins are very sensitive to pH, squid operate at the very peak of blood oxygen utilization, and the resultant Bohr shift would starve the animal of oxygen (Pörtner, 1990, 1994; Fabry et al., 2008; Seibel, 2016). While metabolic suppression was observed in jumbo squid, *Dosidicus gigas*, exposed to 1000 ppm CO_2_, more recent studies have demonstrated no metabolic impacts to adults of bigfin reef squid, *Sepioteuthis lessoniana*, and pygmy reef squid, *Idiosepius pygmaeus*, as well as adults and juvenile *D. gigas*, and *D. pealeii* at equal or greater levels of acidification (Rosa and Seibel, 2008; Hu et al., 2014; Birk et al., 2018; Spady et al., 2019). It is possible that metabolic sensitivity to acidification is life stage dependent in cephalopods with juveniles and adults having the robust physiological machinery needed to manage under OA stress, while the gears of development may be slowed in embryos. Of particular interest, then, is the metabolic scope of the paralarvae under acidification, which are thought to be quite sensitive based on aquaculture studies (pH range of 8.1–8.4 for loliginid paralarvae), and the transition from paralarvae to juvenile in squid (Vidal et al., 2002b).

Observed impacts of acidification to the squid suggest systems of pH and ionic/osmotic balance may be strained, particularly under the severe dosage of 3500 ppm. Zakroff et al. (2019) discussed potential mechanisms of acidification impact to DML and YV, in the scope of a limited energy store and energy budget, suggesting that reductions in growth and YV under hatching are a potential product of upregulation and increased activity of energetically costly proton secreting transporters in ion-transport epithelia (Hu et al., 2010, 2013). The increased proportion of paralarvae showing inflation of the membrane around the eyes under increased OA may further suggest a breakdown in osmoregulatory controls, particularly given the prevalence of ionocytes in the epidermis of cephalopod embryos (Hu et al., 2011). Though it is also plausible that this inflation is related to poorly known osmotic mechanisms that cause the swelling of the egg capsule during development (Hu and Tseng, 2017).

In our sensitive clutches (July 28 and September 14), warming strongly impacted development time, DML and YV, hatching success, and malformations, likely through an increase in metabolic and developmental rates. Warming of +2°C is a standard experimental choice given predicted scenarios under no emission reductions (Pachauri and Meyer, 2014). The warming temperature used, 27°C (+2°C above peak for Vineyard Sound), is within the habitat window reported for *D. pealeii* juveniles and adults, but well above the 23°C maximum reported for egg laying habitats (Jacobson, 2005). Cephalopod eggs typically demonstrate hatching curves wherein success is quite high (>80%) within the preferred thermal window and then drops off rapidly and precipitously above and below certain temperature thresholds (Cinti et al., 2004; Sen, 2005; Staaf et al., 2011; Zeidberg et al., 2011). This threshold was reported to be between 22 and 25°C in *D. opalescens*, but appears to be higher for *D. pealeii* (at least 27°C; Zeidberg et al., 2011). Loss of embryos under warming was primarily in early- and mid-stages of development, suggesting, despite acclimation, a clear and immediate impact to physiology that could easily push embryos past their limits. Disruption of the developmental machinery by warming likely also explains the increased proportion of paralarvae with malformed heads and odd growths, as has been described in *L. vulgaris* (Rosa et al., 2012).

Contrary to some of the observations of temperature and acidification compounding stress effects in *L. vulgaris* embryos and paralarvae, in this work these stressors appear to act antagonistically for most of the factors we measured in the early life stages of *D. pealeii* (Rosa et al., 2014). In part, this antagonism may be due to the much stronger effect size of warming in stress sensitive clutches. In the DML data, for example, warming may have driven embryos to their size floor, the minimum size viable for a paralarvae to hatch, and therefore no further decreases due to acidification could be observed. Previous studies have shown that these two stressors counteract most clearly in hatching time, where warming increases oxygen consumption and developmental rate, while acidification causes developmental delay and potentially metabolic suppression (Pimentel et al., 2012; Rosa et al., 2014; Navarro et al., 2016; Zakroff et al., 2019). In hatching success and malformation, the dampening of acidification impacts is likely driven by a reduction in acidification exposure time as a result of the drastic decrease in time to hatching. The data compiled suggests that warming impacts *D. pealeii* eggs early in development with disruptions, like malformed bodies, potentially propagating to hatching if development doesn’t cease altogether. Acidification, conversely, appears to be a slow burn across development, compounding the buildup of CO_2_ and acidification that would naturally occur due to respiration and thereby causing greater impacts to late stage embryos (Gutowska and Melzner, 2009; Long et al., 2016).

Each clutch of eggs (each trial) demonstrated a different set of responses to acidification and warming across most metrics, particularly DML and YV. As in Zakroff et al. (2019), the comparison of these metrics provides potential insight into the range of physiological coping responses available to *D. pealeii* embryos under multiple stressors (Figure 4). In the June 19 trial, paralarvae from eggs under warming and acidification were slightly smaller in size with substantially less consumed yolk, suggesting a possible overall metabolic suppression that resulted in a relatively resistant clutch. However, the substantive increase in yolk under warming (Figure 2) coincided with an increased loss of embryos in the middle stages of development (Figure 6) and increased premature paralarvae in the hatch (Figure 7). Taken together, these data suggest that the combined stressors, but especially warming, impacted this clutch most during mid-stage development resulting in a notable proportion of unviable hatchlings with too much internal and external yolk left unconsumed, which would be unlikely to survive (Vidal et al., 2002b; Martins et al., 2010; Vidal and Von Boletzky, 2014). July 28 trial showed the reverse, with more yolk consumed in the smaller paralarvae of the combined acidification and warming treatment, suggesting warming outpaced acidification, and the response of the taxed embryos in this case was to consume more yolk in order to cope. The September 14 clutch showed larger paralarvae with slightly more yolk in the control condition, indicating both acidification and warming taxed the energy budgets of these developing embryos. Unfortunately, while it is possible to culture Myopsid paralarvae in aquaria, it is a very challenging proposition for *D. pealeii* that we tried, but could not accomplish (Vidal et al., 2002b). Thus, a question that remains is: given these differential responses to the same stressors between clutches, which strategy would produce the most viable paralarvae in a stressful ocean?

There were two aspects of clutch variability highlighted in these experiments, the first of which is variability between clutches/mothers (which cannot be disentangled from seasonality in our data). Parental conditioning has been shown to impact sensitivities conferred to offspring in fishes and corals (Miller et al., 2012; Putnam and Gates, 2015; Schunter et al., 2016, 2018) Murray et al. (2014) described seasonal pH conditioning of parents in a coastal fish, *Menidia menidia*, which resulted in differential pH sensitivity in offspring. In cephalopods, embryos from winter and summer cohorts of *L. vulgaris* were shown to respond very differently to acidification and warming stress, with summer cohorts being more sensitive (Rosa et al., 2014). Scientists, staff, and fisherman that work with *D*. *pealeii* at the various scientific institutions in Woods Hole, MA, United States anecdotally acknowledge the presence of cohorts within the breeding season, or at least a succession of size classes, but this shift has only roughly been described in the literature as a transition between an early 2-year-old cohort and the new 1-year-old cohort (Arnold et al., 1974; Mesnil, 1977). In Zakroff et al. (2019) sensitivity to acidification started strong and decreased as the season went on, while here, the earliest trial was the most resistant and the latest the most sensitive. This appears to indicate some form of change in parental conferred sensitivity across the 2013 and 2016 seasons from the early summer squid to the early autumn squid, although in opposite directions between these years, which may support the idea of shifting cohorts within the breeding season and suggests that it is parentage rather than seasonality that is driving offspring sensitivity.

The second form of egg clutch variability we examined is variability between the egg capsules of a single mother’s clutch. Even among egg capsules of a single female, squid parentage is a complex proposition since mating can occur with, and sperm can be stored from, multiple males (Buresch et al., 2006). Stress responses and statolith elemental composition have been observed to vary between egg capsules in *D. opalescens* (Navarro et al., 2014, 2016). Ikeda et al. (1999) noted variability within the paralarvae from a single *S. lessoniana* mother, indicating a range in DML correlated with statolith size and hatching time. Our results suggest, at least for DML, that not only does the variation of paralarval sizes within each egg capsule produce an approximately normally distributed curve, but also that egg capsules from the same mother produce very similar distributions (Figure 3). Variability in the sample of hatchling DML has been noted previously as a possible consequence of egg position within the egg capsule and, in a natural setting, of egg capsule within the egg mass, both of which can impact oxygen availability and thus developmental rate and ultimately paralarval size and yolk content (Steer and Moltschaniwskyj, 2007; Vidal and Von Boletzky, 2014). Under additional stressors, these distributions appear to shift. Relatively light stress appears to simply shift the distributions, while heavier stressors cause flattening and spreading of these curves (Figure 3). It has been theorized that size variation among offspring acts as a kind of adaptation to unpredictable environments, with selection pressures (in this case increased environmental stress) acting upon both the mean and variance of an offspring distribution (Marshall et al., 2008).

In cephalopods, paralarval size is, on a taxonomic level, known to correlate to egg size (Laptikhovsky et al., 2013). Adult squid do not retain lipid reserves, so maternal investment in reproduction is primarily driven by the allocation of resources between somatic vs. reproductive growth (Pecl and Moltschaniwskyj, 2006). Energy for egg production is captured through recent feeding, so while successive clutches of eggs may degrade in quality as the state of the mother degrades, maternal input within a clutch may, as appears to be supported by our DML evidence, be relatively consistent across egg capsules (Steer et al., 2004). It is plausible then that the breakdown of similarities between egg capsules under stress could owe to differences in genetic background due to paternity, though this is purely speculative without much more robust experimentation.

This study demonstrated that *D. pealeii* embryos and paralarvae reared under severe, chronic acidification and warming could show a range of responses from sensitive to resistant. These responses are driven by between clutch differences, which are likely representations of parentage, but may also be influenced by seasonality. Responses are also variable given the complexity of interacting and antagonistic physiological processes influenced by warming and acidification in this system. These experiments were limited in a number of key ways. As an in lab experiment, factors of flow, egg capsule density, and variability that occur in the natural system are not represented here. Variability of pH in natural systems is thought to decrease impacts in some organisms by reducing exposure time, which appears to be an important factor in acidification’s impact on *D. pealeii* eggs (Shaw et al., 2013). While a growing body of literature is beginning to suggest squid, at least embryos and adults (there is still a great deal left to understand with respect to paralarvae), may be fairly robust in the face of OA, these responses may be taxon, population, or region specific making it difficult to generalize (Kroeker et al., 2017; Birk et al., 2018; Spady et al., 2019). Warming, however, clearly has its limits in *D*. *pealeii* embryonic development, but squid have the advantage of mobility in coping with that (Doubleday et al., 2016). A fecund, plastic, year class species, such as *Doryteuthis pealeii*, appears well suited to rapid adaptability under rapid global ocean change. It is important, therefore, to continue to describe the signs and understand the mechanisms of that adaptability, and to investigate its limits, in order to inform how we design experiments to diagnose sensitivity and adaptability in other marine taxa.

## Data Availability Statement

The datasets generated for this study are available on request to the corresponding author.

## Ethics Statement

Ethical guidelines for animal research are institutionally specific in the United States and research involving cephalopods does not require ethical review or approval from the Woods Hole Oceanographic Institution’s IACUC. Transport, care, and treatment of the cephalopods used here was done following international guidelines in the literature, particularly those which have since been summarized in Fiorito et al. (2015).

## Author Contributions

CZ and TM designed the experiments. CZ performed the experiments and collected the data. The data analysis and statistics was performed by CZ with assistance from TM. CZ wrote the first draft of the manuscript. CZ and TM revised the manuscript. Both authors read and approved the submitted version of the manuscript.

## Conflict of Interest

The authors declare that the research was conducted in the absence of any commercial or financial relationships that could be construed as a potential conflict of interest.

## Abbreviations

DML: dorsal mantle length
ESL: Environmental Systems Laboratory
KW: Kruskal–Wallis test
LR: linear regression
MBL: Marine Biological Laboratories
OA: ocean acidification
YV: yolk sac volume.
