# Supplementary material for: Antagonistic Interactions and Clutch-Dependent Sensitivity Induce Variable Responses to Ocean Acidification and Warming in Squid (Doryteuthis pealeii) Embryos and Paralarvae
Source: Front Physiol. 2020 May 20;11:501. doi: 10.3389/fphys.2020.00501 (PMC7251416; doi:10.3389/fphys.2020.00501)
Supplement: Supplementary file 1 [file Data_Sheet_1.PDF]

Table S1. Three-way Type II ANOVAs for dorsal mantle length and (log-transformed) yolk volume data, for each trial and compiled across trials. Significant p values (α = 0.05) in bold. '<<' indicates a p-value with a negative exponent of 50 or greater.

	Mantle Length					Yolk Sac Volume				
Source	SS	df	*F*	*P*	ω^2	SS	df	*F*	*P*	ω^2
**Jun 19**										
Temp	0.011	1	0.728	0.394	-0.000	165.9	1	326.3	**<<0.001**	0.410
pCO_2	2.146	2	69.37	**<0.001**	0.217	18.52	2	18.21	**<0.001**	0.043
Temp : pCO_2	0.064	2	2.059	0.129	0.003	13.86	2	13.63	**<0.001**	0.032
Residual	7.516	486				204.9	403			
**Jul 28**										
Temp	0.887	1	52.11	**<0.001**	0.077	1.780	1	9.091	**0.002**	0.020
pCO_2	1.514	2	44.46	**<0.001**	0.132	1.358	2	3.466	**0.032**	0.012
Temp : pCO_2	0.362	2	10.62	**<0.001**	0.029	3.942	2	10.06	**<0.001**	0.045
Residual	8.460	497				71.87	367			
**Sep 14**										
Temp	2.203	1	256.4	**<0.001**	0.252	9.148	1	59.41	**<0.001**	0.109
pCO_2	1.453	2	84.52	**<0.001**	0.165	1.928	2	6.273	**0.002**	0.020
Temp : pCO_2	0.800	2	46.51	**<0.001**	0.090	3.341	2	10.87	**<0.001**	0.037
Residual	4.237	493				68.25	444			
**Compiled**										
Trial	10.32	2	376.6	**<<0.001**	0.257	352.2	2	619.5	**<<0.001**	0.383
Temperature	2.148	1	156.9	**<0.001**	0.054	41.59	1	146.3	**<0.001**	0.045
pCO_2	4.768	2	174.1	**<<0.001**	0.119	7.062	2	12.42	**<0.001**	0.008
Trial : Temp	1.056	2	38.52	**<0.001**	0.026	140.2	2	246.7	**<<0.001**	0.153
Trial : pCO_2	0.408	4	7.443	**<0.001**	0.010	11.93	4	10.50	**<0.001**	0.013
Temp : pCO_2	0.782	2	28.54	**<0.001**	0.019	6.433	2	11.32	**<0.001**	0.007
Trial : Temp : pCO_2	0.443	4	8.093	**<0.001**	0.011	14.71	4	12.94	**<0.001**	0.016
Residual	20.21	1476				345.1	1214			

Table S2. Hatching success distributions and G-tests. Counts of staged, failed embryos and paralarvae, presented by trial and treatment. Staged embryos were categorized using Arnold et al. (1974) as Early (embryonic stages 1 - 16), Middle (17 - 26), or Late (27 - 30). G-tests are reported with significant p-values ($p < 0.05$) in bold. Exponents of the p-value are listed to compare degrees of significance among comparisons with p-values less than 0.001.

Temperature (ºC)	pCO_2 (ppm)	Early	Middle	Late	Hatched	Percent Hatched	Source	*G*	df	p	exp
**Jun 19**											
20	400	1	1	1	273	98.9	400 X 2200	22.01	3	**<0.001**	-05
	2200	5	0	20	310	92.7	400 X 3500	90.45	3	**<0.001**	-19
	3500	2	2	48	132	73.2	2200 X 3500	45.16	3	**<0.001**	-10
							All 20 ºC	100.6	6	**<0.001**	-19
27	400	5	4	4	314	96.1	400 X 2200	158.4	3	**<0.001**	-34
	2200	9	105	18	190	59.2	400 X 3500	27.45	3	**<0.001**	-06
	3500	7	6	22	183	84.1	2200 X 3500	87.82	3	**<0.001**	-19
							All 27 ºC	205.0	6	**<0.001**	-41
20 X 27							400 X 400	5.313	3	0.150	-01
							2200 X 2200	175.7	3	**<0.001**	-38
							3500 X 3500	20.34	3	**<0.001**	-04
**Jul 28**											
20	400	3	1	19	473	95.4	400 X 2200	20.21	3	**<0.001**	-04
	2200	3	0	2	562	99.1	400 X 3500	10.77	3	**0.013**	-02
	3500	0	0	28	323	92.1	2200 X 3500	45.10	3	**<0.001**	-10
							All 20 ºC	48.08	6	**<0.001**	-08
27	400	135	45	59	144	34.0	400 X 2200	317.5	3	**<0.001**	-86
	2200	1	3	18	330	93.2	400 X 3500	134.4	3	**<0.001**	-68
	3500	10	19	93	165	54.7	2200 X 3500	126.4	3	**<0.001**	-29
							All 27 ºC	414.1	6	**<0.001**	-27
20 X 27							400 X 400	408.4	3	**<0.001**	-88
							2200 X 2200	30.20	3	**<0.001**	-06
							3500 X 3500	122.7	3	**<0.001**	-26
**Sep 14**											
20	400	71	0	0	385	85.1	400 X 2200	257.6	3	**<0.001**	-55
	2200	21	7	119	181	57.8	400 X 3500	179.0	3	**<0.001**	-39
	3500	10	0	85	275	74.2	2200 X 3500	36.37	3	**<0.001**	-08
							All 20 ºC	299.9	6	**<0.001**	-62
27	400	15	2	4	346	94.0	400 X 2200	3.524	3	0.318	-01
	2200	21	0	4	375	93.8	400 X 3500	172.3	3	**<0.001**	-37
	3500	165	4	13	231	56.8	2200 X 3500	171.6	3	**<0.001**	-37
							All 27 ºC	252.0	6	**<0.001**	-51
20 X 27							400 X 400	40.37	3	**<0.001**	-09
							2200 X 2200	206.9	3	**<0.001**	-44
							3500 X 3500	232.1	3	**<0.001**	-50

Table S3. Malformation distributions and G-tests. Counts of normal and malformed paralarvae, presented by trial and treatment. Malformed paralarvae were categorized as Premature (external yolk remaining), Eye Bulge (inflated eye membrane), or Malformed Head (malformations/growths on head and mantle). G-tests are reported with significant p-values ($p < 0.05$) in bold. Exponents of the p-value are listed to compare degrees of significance among comparisons with p-values less than 0.001.

Temperature (ºC)	pCO_2 (ppm)	Normal	Premature	Eye Bulge	Malformed Head	Source	*G*	df	p	exp
**Jun 19**										
20	400	168	6	2	2	400 X 2200	12.51	3	**0.006**	-03
	2200	190	20	12	1	400 X 3500	139.7	3	**<0.001**	-30
	3500	27	43	21	5	2200 X 3500	99.8	3	**<0.001**	-21
						All 20 ºC	155.7	6	**<0.001**	-31
27	400	179	4	3	18	400 X 2200	25.15	3	**<0.001**	-05
	2200	61	7	8	21	400 X 3500	101.7	3	**<0.001**	-22
	3500	77	34	39	16	2200 X 3500	25.85	3	**<0.001**	-05
						All 27 ºC	112.4	6	**<0.001**	-22
20 X 27						400 X 400	13.90	3	**0.003**	-03
						2200 X 2200	48.29	3	**<0.001**	-10
						3500 X 3500	18.73	3	**<0.001**	-04
**Jul 28**										
20	400	202	13	8	3	400 X 2200	22.77	3	**<0.001**	-05
	2200	260	28	48	9	400 X 3500	137.2	3	**<0.001**	-29
	3500	46	63	21	14	2200 X 3500	105.8	3	**<0.001**	-23
						All 20 ºC	166.8	6	**<0.001**	-33
27	400	121	25	6	1	400 X 2200	15.49	3	**0.001**	-03
	2200	148	20	31	1	400 X 3500	61.25	3	**<0.001**	-13
	3500	45	54	17	12	2200 X 3500	73.33	3	**<0.001**	-16
						All 27 ºC	99.58	6	**<0.001**	-19
20 X 27						400 X 400	11.57	3	**0.009**	-03
						2200 X 2200	4.485	3	0.214	-01
						3500 X 3500	0.338	3	0.953	-01
**Sep 14**										
20	400	234	8	10	3	400 X 2200	30.94	3	**<0.001**	-07
	2200	125	14	31	1	400 X 3500	241.1	3	**<0.001**	-52
	3500	48	98	44	7	2200 X 3500	111.9	3	**<0.001**	-24
						All 20 ºC	266.6	6	**<0.001**	-54
27	400	210	7	0	0	400 X 2200	44.30	3	**<0.001**	-09
	2200	161	18	20	4	400 X 3500	216.0	3	**<0.001**	-46
	3500	55	64	41	11	2200 X 3500	89.74	3	**<0.001**	-19
						All 27 ºC	227.3	6	**<0.001**	-46
20 X 27						400 X 400	16.32	3	**<0.001**	-04
						2200 X 2200	6.622	3	0.085	-02
						3500 X 3500	6.829	3	0.078	-02

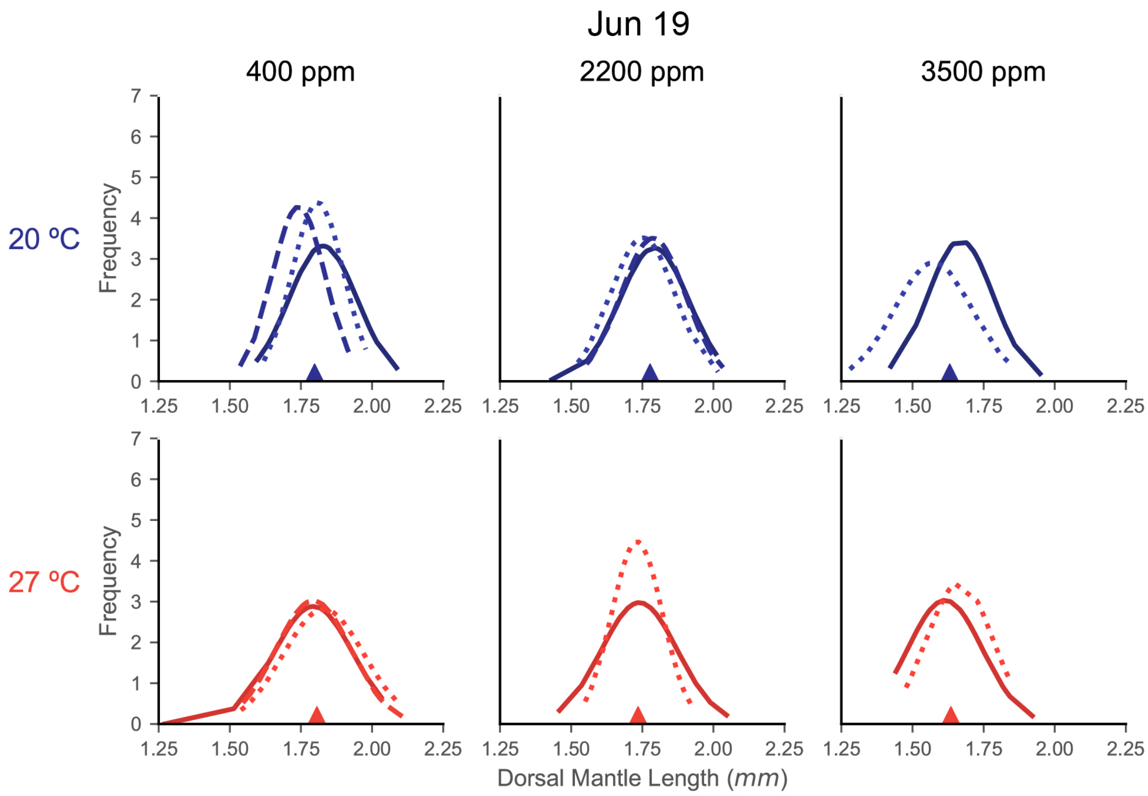

Figure S1. Distributions of mantle length from individual egg capsules of the June 19 trial. Fitted normal distributions of dorsal mantle length histogram density from individual egg capsules in each treatment of the June 19 trial. Histograms are not shown for clarity of curves, but DML data were segmented in 0.05 mm bins. Each plot is a treatment combination, with column determining acidification (titles) and row determining temperature treatment (also differentiated by color: top/blue = 20 ºC, bottom/red = 27 ºC). Each line represents the curve from the sampling of an individual egg capsule for dorsal mantle length (x-axis). Lines are shaded and patterned to help differentiate individual egg capsules within a plot, but this carries no relationship or meaning across the plots. The filled triangle on the x-axis marks the mean value for the compiled sample of that treatment.

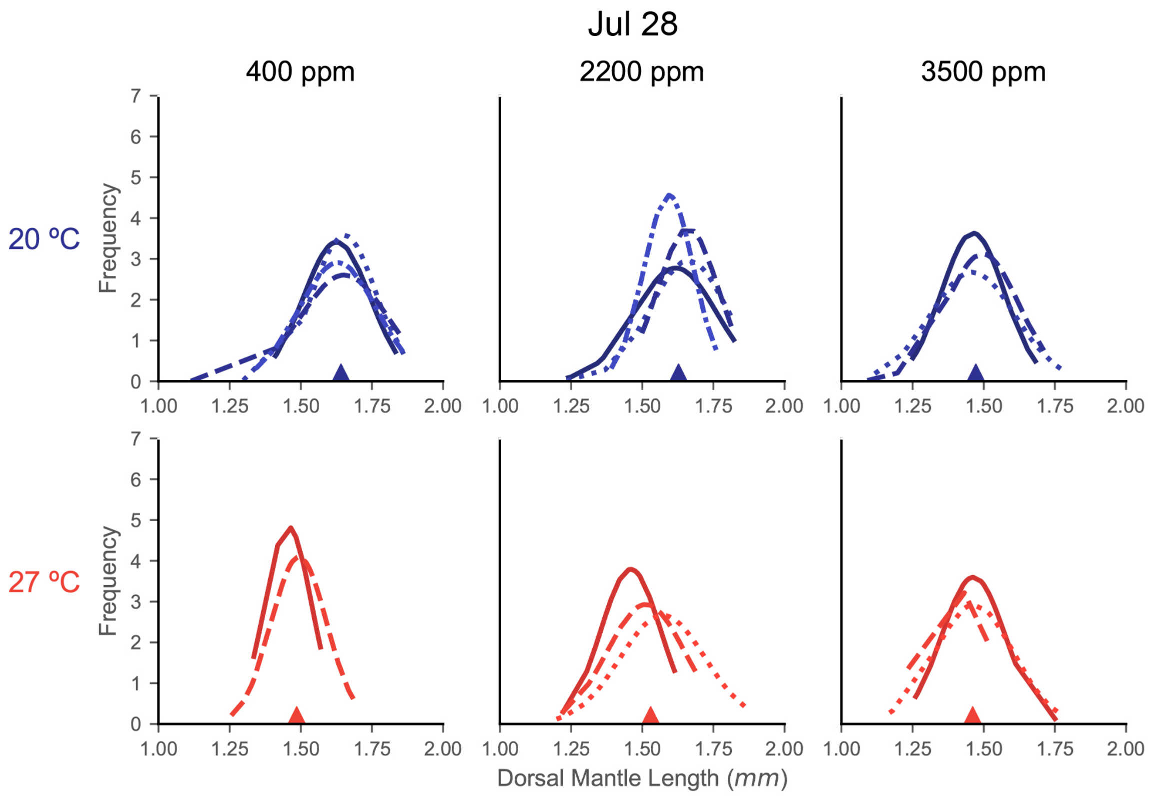

Figure S2. Distributions of mantle length from individual egg capsules of the July 28 trial. Fitted normal distributions of dorsal mantle length histogram density from individual egg capsules in each treatment of the July 28 trial. Histograms are not shown for clarity of curves, but DML data were segmented in 0.05 mm bins. Each plot is a treatment combination, with column determining acidification (titles) and row determining temperature treatment (also differentiated by color: top/blue = 20 ºC, bottom/red = 27 ºC). Each line represents the curve from the sampling of an individual egg capsule for dorsal mantle length (x-axis). Lines are shaded and patterned to help differentiate individual egg capsules within a plot, but this carries no relationship or meaning across the plots. The filled triangle on the x-axis marks the mean value for the compiled sample of that treatment.
